# In-silico Exploration of Channel Type and Efflux Silicon Transporters and Silicification Proteins in 80 Sequenced Viridiplantae Genomes

**DOI:** 10.3390/plants9111612

**Published:** 2020-11-20

**Authors:** Muhammad Amjad Nawaz, Farrukh Azeem, Alexander Mikhailovich Zakharenko, Xiao Lin, Rana Muhammad Atif, Faheem Shehzad Baloch, Ting-Fung Chan, Gyuhwa Chung, Junghee Ham, Sangmi Sun, Kirill S. Golokhvast

**Affiliations:** 1 Laboratory of Bio-Economics and Biotechnology, Department of Bio-Economics and Food Safety, School of Economics and Management, Far Eastern Federal University, 690950 Vladivostok, Russia; amjad_ucauos@yahoo.com; 2 Department of Bioinformatics and Biotechnology, Government College University, Faisalabad 38000, Pakistan; farrukh@gcuf.edu.pk; 3 N.I. Vavilov All-Russian Institute of Plant Genetic Resources, 42, 44 Bolshaya Morskaya Street, 190000 St. Petersburg, Russia; rarf@yandex.ru; 4 Center for Soybean Research, State Key Laboratory of Agrobiotechnology, The Chinese University of Hong Kong SAR, Hong Kong 999077, China; alanlamsiu@gmail.com (X.L.); tf.chan@cuhk.edu.hk (T.-F.C.); 5 US-Pakistan Centre for Advanced Studies in Agriculture and Food Security, University of Agriculture, Faisalabad 38040, Pakistan; dratif@uaf.edu.pk; 6 Faculty of Agricultural Sciences and Technologies, Sivas University of Science and Technology, Sivas 58140, Turkey; balochfaheem13@gmail.com; 7 Department of Biotechnology, Chonnam National University, Chonnam 59626, Korea; chung@chonnam.ac.kr; 8 Department of Health Policy and Management, Wonkwang University, Iksan, Jeonbuk 54538, Korea; hamssine@hamssine.co.kr; 9 Education and Scientific Center of Nanotechnology, Far Eastern Federal University, 690950 Vladivostok, Russia; 10 Pacific Geographical Institute, FEB RAS, 7 Radio street, 690014 Vladivostok, Russia

**Keywords:** biosilicification, channel type Si transporter, influx transporter, NIPs, silica accumulation, Siliplant 1, Si efflux, Viridiplantae

## Abstract

Silicon (Si) accumulation protects plants from biotic and abiotic stresses. It is transported and distributed within the plant body through a cooperative system of channel type (e.g., *OsLsi1*) and efflux (Lsi2s e.g., *OsLsi2*) Si transporters (SITs) that belong to Noduline-26 like intrinsic protein family of aquaporins and an uncharacterized anion transporter family, respectively. Si is deposited in plant tissues as phytoliths and the process is known as biosilicification but the knowledge about the proteins involved in this process is limited. In the present study, we explored channel type SITs and Lsi2s, and siliplant1 protein (Slp1) in 80 green plant species. We found 80 channel type SITs and 133 Lsi2s. The channel type SITs characterized by the presence of two NPA motifs, GSGR or STAR selectivity filter, and 108 amino acids between two NPA motifs were absent from Chlorophytes, while Streptophytes evolved two different types of channel type SITs with different selectivity filters. Both channel type SITs and Lsi2s evolved two types of gene structures each, however, Lsi2s are ancient and were also found in Chlorophyta. Homologs of Slp1 (225) were present in almost all Streptophytes regardless of their Si accumulation capacity. In Si accumulator plant species, the Slp1s were characterized by the presence of H, D-rich domain, P, K, E-rich domain, and P, T, Y-rich domain, while moderate Si accumulators lacked H, D-rich domain and P, T, Y-rich domains. The digital expression analysis and coexpression networks highlighted the role of channel type and Lsi2s, and how Slp1 homologs were ameliorating plants’ ability to withstand different stresses by co-expressing with genes related to structural integrity and signaling. Together, the in-silico exploration made in this study increases our knowledge of the process of biosilicification in plants.

## 1. Introduction

Silicon (Si) exerts beneficial effects on the growth and productivity of plants and has been recognized as a beneficial element by the International Plant Nutrition Institute [1]. Decades of research on Si have authenticated its importance towards plant’s ability to withstand biotic and abiotic stresses [2]. Si accumulation in plants is regarded as beneficial regardless of their Si accumulation capacity; when deprived of Si, plants are susceptible to stresses as compared to the ones grown in Si. In soil, Si is present as monosilicic acid [Si(OH)_4_] at pH 9 with variable concentrations based on soil type [3]. Silicic acid is taken up through a cooperative system of channel type Si transporters (SITs) and efflux silicon transporters (homologs of *OsLsi2*, from now on Lsi2s) [4]. The identification of these SITs in rice i.e., *OsLsi1* and *OsLsi2* was a breakthrough, which led to a substantial understanding of Si transport in plants [5,6]. The first identified channel type SIT (*OsLsi1*) is an influx SIT and a member of Noduline-26-like intrinsic proteins (NIPs) [5]. Functional characterization of *OsLsi1* homologs in different plant species have enabled us to understand that the Si specificity is associated with two highly conserved NPA motifs (Asn-Pro-Al) and an aromatic/arginine (ar/R) region formed by four residues which function as selectivity filter (SF). The Lsi1s have characteristic SFs i.e., Gly-Ser-Gly-Arg (GSGR) or Ser-Thr-Ala-Arg (STAR) [7,8]. In addition, a precise spacing of 108 amino acids (AAs) (or 109 AAs according to [9]) between the two NPA motifs is essential for Si permeability in plants [10]. These channel type SITs facilitate the transport of Si across the plasma membrane between the external solution (i.e., apoplast) and the plant cells. After entering in the cell, Si moves further across the same cell (endodermis) or different cell types (epidermal, hypodermal, and cortical cells) by Lsi2, depending on the plant species [4,6]. The rice Lsi2 (*OsLsi2*) has 11 predicted transmembrane domains (TMDs) and it belongs to an uncharacterized anion transporter family. It lacks homology with channel type SIT and limited studies have described any conserved characteristics within the characterized as well as predicted Lsi2s [2,11]. The cooperative system of both channel type SITs and Lsi2s enable plants to translocate Si to the aerial parts of the plants where it is deposited as amorphous Si(SiO_2_) [12].

The amorphous Si deposits are defined as phytoliths [13] and the process of deposition is called biosilicification [14]. Si is deposited in different locations within plants i.e., root endodermis, leaf epidermis, and abaxial epidermis of inflorescence bracts where it can accumulate as phytoliths within the cell lumen or directly laid down on the cell wall matrix; cell wall matrix provides structural template on which Si can be laid down [12,15]. The process of biosilicification crosses kingdoms of living organisms and can be found in diverse eukaryotic lineages [16]. Recent studies have demonstrated that SITs appeared in plants due to the selectivity shift allowing subfunctionalization of ancestral NIPs [17]. Furthermore, the bacterial arsenic Lsi2s are the source of modern-day seed plant nutrient transporters. Therefore, the process of biosilicification is ancient [16] and has evolved in diverse kingdoms and lower phylogenetic clads differently. Although many studies explored the evolution of biosilicification in relevance to plants [18], limited studies attempted to explore the proteins that are responsible for biosilicification upon the arrival of Si at depositing sites within plant [19]. However, contrary to plants, the process of biosilicification is well understood in diatoms [20]. The functional characterization of biosilicification-related proteins in diatoms did not help much in the identification of proteins involved in this process because of no sequence homology between plant and diatom silicification proteins [21,22]. So far, the research in nonplant lineage biosilicification has demonstrated that the silicification related proteins must have certain features such as proline, lysine, and glutamine-acid-rich (P, K, E-rich) domain(s), RXL domain [23,24], and several proline residues [25]. These characteristics and the developments in RNA sequencing have recently aided the characterization of a Siliplant1 (Slp1) protein in sorghum [19].

Developments in genomics and next generation sequencing are rapidly increasing the available plant genomes in public repositories [26]. These developments allow us to identify homologs and fill the missing links in different pathways such as biosilicification. With the confirmation that NIPs and arsenite transporters having essential signatures can transport Si across membranes, we report the discovery of SITs in 80 sequenced viridiplantae genomes. Additionally, we present the first large scale dataset of putative Slps in plants. This effort will allow a precise understanding of the biosilicification process in plants and will greatly supplement the studies related to evolution of exploration of biosilicification.

## 2. Material and Methods

### 2.1. Data Retrieval

*Oryza sativa* channel type SIT *OsLsi1* (*LOC_Os02g51110*), efflux Si transporter *OsLsi2* (*LOC_Os03g01700*), and *Sorghum bicolor* siliplant1 (*SbSlp1*, *Sobic.001G266300*) were used as query sequences for BlastP 2.2.28+ searches using the default parameters against 80 Viridiplantae genomes available from Phytozome v12.1; Joint Genome Institute (JGI; https://www.phytozome.net) [27]. The 80 species belonged to all major phylogenetic clads of Viridiplantae (Table 1). All the retrieved results were collected and manually curated to produce a nonredundant dataset that was then subjected to subsequent analysis.

### 2.2. Sequence Analyses

The retrieved sequences were aligned in MEGA X using built-in MUSCLE. For channel type SITs, the SF and NPA motif amino acids (AAs) were manually searched against *OsLsi1*. The distance between the 2 NPA motifs was calculated. Transmembrane domains (TMDs) were predicted by TMHMM server (cbs.dtu.dk/services/TMHMM/; [28]). Protein molecular weight (Mw) and theoretical isoelectric point (pI) were computed in protein identification and analysis tools on the ExPASy Server (https://web.expasy.org/compute_pi/) [29]. Amino acid frequencies were calculated in TargetP 1.1 Server [30]. Protein subcellular localization was predicted in TargetP-2.0 Server [30]. Repeats in peptide sequences were predicted in RADAR (ebi.ac.uk/Tools/pfa/radar/) [31]. Conserved motifs were found in the peptide sequences using MEME tool (meme.nbcr.net/meme/tools/meme) [32]. SignalP was used to predict subcellular localization and cleavage sites [33].

### 2.3. Phylogenetic Analyses

Separate alignments for channel type SITs, Lsi2s, and Slp1s were generated using MUSCLE in MEGA X [34]. The alignments were submitted to IQ-TREE (http://www.cibiv.at/software/iqtree) with default parameters for tree inference [35]. ModelFinder [36] was used to find the best fit model according to the Bayesian information criterion with ultrafast bootstrap [37]. The trees were visualized and edited in iTOL (http://itol.embl.de/ [38]). Estimates of evolutionary divergence between sequences were computed in MEGA X using the Poisson correction model [39].

### 2.4. Prediction of Silica Precipitation Potential of Selected Peptide Sequences

Selected peptide sequences (repeats) were submitted to protein secondary structure ROSETTA server which uses consensus results from 3 programs, including HHpred, RaptorX, and SPARKS [40]. The generated peptide models were used to study alanine scanning and molecular interactions with Si(OH)_3_O^−^. Ligplot v.4.5.3 software implemented in MOLECULAR OPERATING ENVIRONMENT (MOE) were used for prediction of this interactions [41]. The figures were generated in Icn3D v 2.19.0 and ChemWindow V6.0 (Bio-Rad Laboratories).

### 2.5. Digital Expression and Co-Expression Network Analysis

The digital expression comparison and graphical presentation for selected genes in rice, soybean, and tomato was performed in ‘eFP browser’ of BAR tools (The Bio-Analytic Resource for Plant Biology, http://bar.utoronto.ca/) [42,43]. Coexpression network comparison was performed with Network comparer tool in PlaNet (http://aranet.mpimp-golm.mpg.de/index.html; [44]).

## 3. Results

### 3.1. Identification of SITs and Slps

#### 3.1.1. Channel Type SITs in Viridiplantae

**Figure 1:**
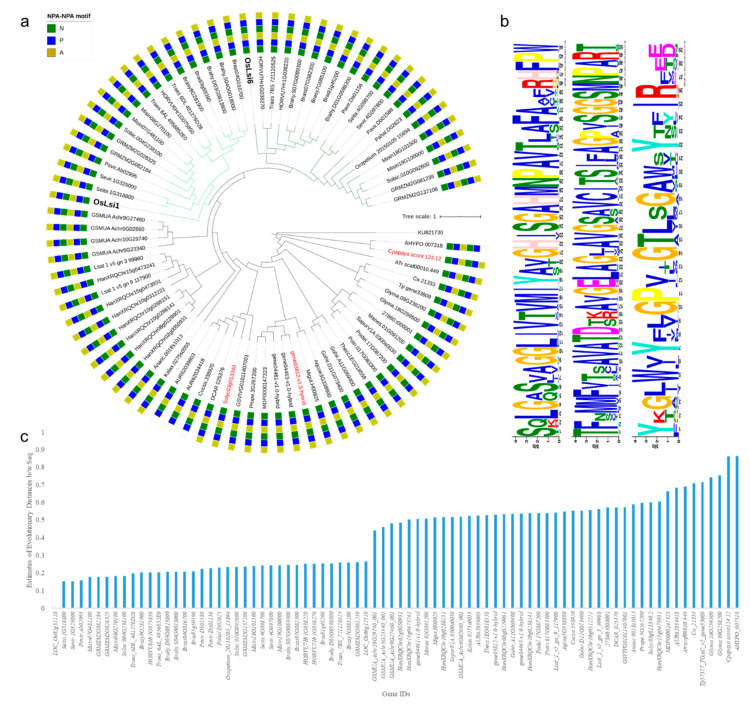
(**a**) Maximum-likelihood tree of *OsLsi1* homologs (80) in studied viridiplantae genomes. *KU821730* (*Spongosphaera streptacantha* SIT-L gene) was used as an outgroup. The sequences were then aligned by MUSCLE in MEGA X and exported to IQ-Tree. The tree was generated using substitution model JTT + I + G4 as a model of rate heterogeneity and Ultrafast Bootstrap with 1000 replicates. The red colored genes have 109 AAs between the NPA motifs. The empty golden square represents the presence of AA other than Ala i.e., Val. The light green clad color shows *OsLsi1* subclad, the dark green color shows *OsLsi6* subclad. (**b**) Conserved motifs present in plant channel type SITs. (**c**) Estimation of evolutionary divergence between *OsLsi1* and the identified genes.

Using the characterized *OsLsi1* as a query sequence, BLAST searches against 80 plant genomes resulted in 1158 homologs belonging to 78 species (Supplementary Table S1). The homologs were screened on the basis of BlastP 2.2.28+ searches using the default parameters in Phytozome. The criteria used to screen the database for homologs/candidate genes may have limited the detection of poorly annotated, or truncated sequence reads. These putative channel type SITs belonged to aquaporin NIP gene family or uncharacterized proteins. The screening for STAR or GSGR SF and two NPA motifs resulted in 80 genes [10]. In total, 3 genes had 109 AAs between both NPA motifs while the rest had 108 AAs. The channel type SITs were characterized by the presence of five or six TMDs similar to *OsLsi1* [5]. One *F. vesca* gene (*gene03812-v1.0-hybrid*) had 10 TMDs. A very small number of genes had variation in their second NPA motif where Ala (A) was replaced by Val (V) (Figure 1a). In total, 1 of 80 genes had STAR SF (*AHYPO_007318*). It was reported earlier that a P-to-L mutation in pumpkin causes miss-localization of channel type SITs into endoplasmic reticulum [45] but this mutation was not found in the 80 channel type SITs.

The phylogenetic tree showed that the monocots formed a separate clad, which was divided into two subclads. Members of the two subclads were grouped with *OsLsi1* and *OsLsi6*, respectively. The monocot specific clad contained four banana homologs that grouped as a parent branch in this clad. The gene containing STAR SF did not group with the rest of the channel type SITs except one papaya gene (*gene03812-v1.0-hybrid*) but both genes had different SF. The homologs in Fabales (soybean, red clover, and chickpea) clustered together. Similarly, genes belonging to Chenopodiaceae and Malvaceae grouped together. Interestingly, our study involved two Caryophyllales i.e., *C. quinoa* (Chenopodiaceae) and *A. hypochondriacus* (Amaranthaceae), which resulted in one candidate gene from each species but had different SF i.e., GSGR and STAR, respectively (Figure 1a). Other homologs from *C. sativus* (Cucurbitales)*, R. communis, P. deltoids, P. trichocarpa* (Malpighiales)*, S. lycopersicum* (Solanales)*, M. domestica, P. persica* (Rosales)*, V. vinifera* (vitales)*, A. coerulea* (Ranunculales), *M. guttatus* (Lamiales), *C. papaya* (Brassicales), *D. carota* (Apiales), *S. purpurea* (Ericales) *M. esculanta (mushroom)* formed a separate clad. We identified three highly conserved motifs in 80 channel type SITs (Figure 1b). Furthermore, previous studies reported that there could be a correlation between the sequence similarity and Si transport capacity [11]. In this regard, we computed the estimates of evolutionary divergence between sequences. Interestingly, it was found that the sequences from known Si accumulators had higher similarity with *OsLsi1* (Figure 1c).

#### 3.1.2. Lsi2s in Viridiplantae

**Figure 2:**
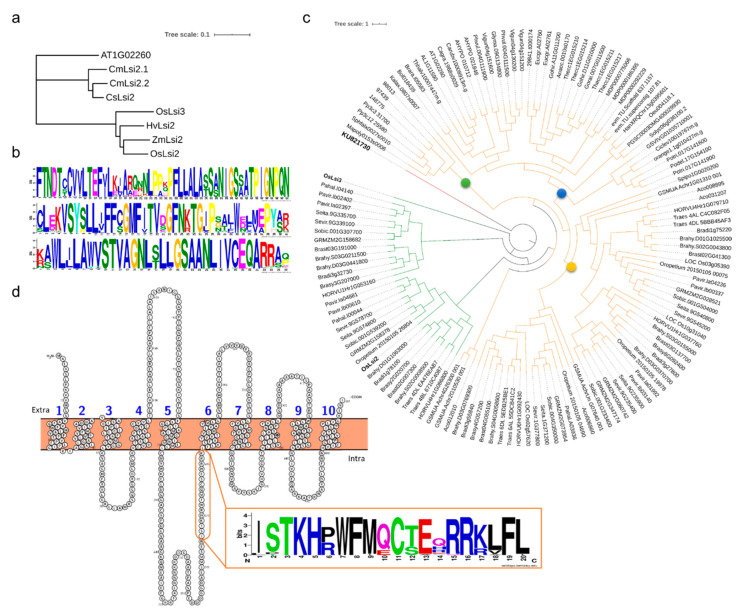
(**a**) Neighbor-joining tree of Lsi2s that have been characterized. (**b**) Conserved motifs found in all *OsLsi2* homologs, and (**c**) ML tree of Lsi2s in studied viridiplantae genomes. *KU821730* (*Spongosphaera streptacantha* SIT-L gene) was used as an outgroup. The sequences were then aligned by MUSCLE in MEGA X and exported to IQ-Tree. The tree was generated using substitution model VI + I + G4 (Invar + Gamma with 4 categories) as a model of rate heterogeneity and Ultrafast Bootstrap with 1000 replicates. (**d**) *OsLsi3* gene showing TMDs (prepared in PROTER V 1.1 with default settings) [47]. The orange circle highlights the highly conserved region. The conserved region is represented by a sequence logo (prepared in WebLogo [48]).

The BlastP search using *OsLsi2* as a query against 80 viridiplantae genomes resulted in the identification of 255 genes. To screen the *OsLsi2* gene homologs, first we searched the known Lsi2s in rice (*OsLsi2* and *OsLsi3*), maize (*ZmLsi2*), cucumber (*CsLsi2*), barley (*HVLsi2*), and pumpkin (*CmLsi2-1* and *CmLsi2-2*) to visualize any conserved sequences in them [46] (Figure 2a). The alignment showed that the *OsLsi2* residues between 212 to 343 were highly variable and the rest were conserved regions (Supplementary Figure S1a). By using MEME tool, we found three highly conserved motifs (Supplementary Figure S1b). According to a previous report, the Lsi2s contain 9–11 TMDs [46] and in the current study we found 133 genes having at least 9 TMDs. These 133 genes contained the three conserved motifs present in known Lsi2s (Figure 2b). The pI ranged from 4.87 to 9.41 and the Mw ranged from 8457.98 kDa to 101310.2 kDa. One gene i.e., *AHYPO_010712* had the highest Mw (114798.26 kDa) and the largest number of TMDs i.e., 18 (Supplementary Table S2).

The maximum-likelihood (ML) phylogenetic tree formed one major clad rooted on one gene i.e., *OsLsi3,* which further divided the genes into clads (Figure 2c). The first two clads (shown in green) consisted of members of Poales (*Poaceae*), while, all other genes formed a separate clad (shown in orange). The genes belonging to early plant lineages i.e., *P. patens*, *S. moellendorffii*, *M. polymorpha*, and *S. fallax* were grouped together (represented by a green circle). The remaining genes grouped in a major clad forming two subclads i.e., monocot specific (represented by a dark yellow circle) and dicot specific (represented by a blue circle) (Figure 2c). Since some monocot genes formed a separate subclad with dicots, therefore, we wanted to know if there is any distinct motif present within the genes that formed clad with known rice Lsi2s (shown in green). We found that these genes had a much-conserved motif i.e., XXTKHXWFXXCXXXXRX (Figure 2d). This highly conserved region is located in rice Lsi2s exactly before the sixth TMD. This region was highly variable in all early lineage, dicot, and remaining monocot homologs. Since, the Equisetum (*Equisetum arvense*) Lsi2s i.e., *EaLsi2*s, and *CmLsi2*s did not contain this motif, therefore, based on the studies conducted in homologs of Lsi2 in different plant species, it could be suggested that it might have no functional importance. A further characterization study of this region could increase our understanding in this regard. It suggested a possibility of differential evolution for these Lsi2s. For further confirmation, we introduced the peptide sequences of *EaLsi2-1* and *EaLsi2-2* and prepared the ML tree where *EaLsi2*s grouped with ancestral plant lineages instead of rice or other dicots (Supplementary Figure S2).

#### 3.1.3. Siliplant1 Homologs in Viridiplantae

**Figure 3:**
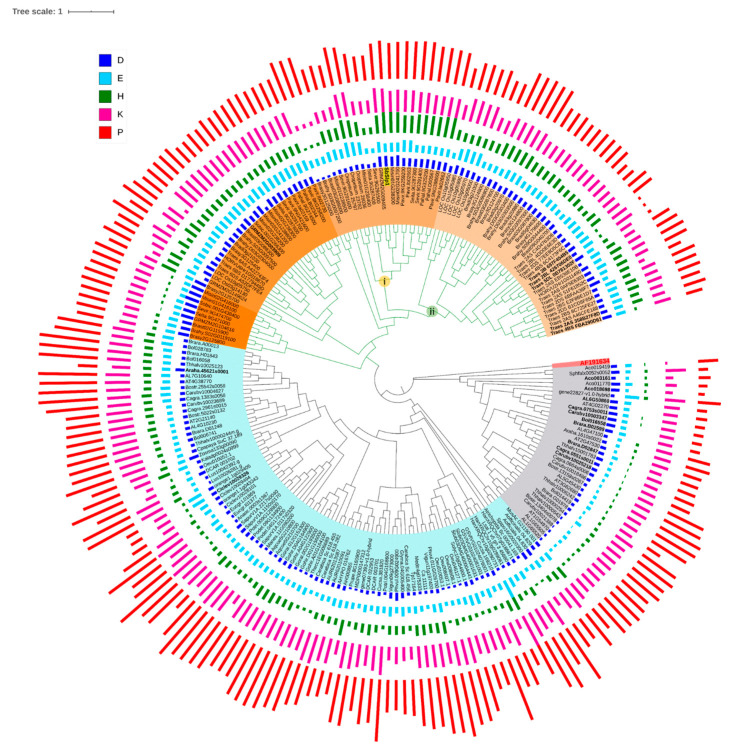
Maximum-likelihood tree of *SbSlp1* homologs in viridiplantae. *Cylindrotheca fusiformis* silaffin precursor protein (*sil1p, AF191634*) gene was used as an outgroup. The sequences were aligned by MUSCLE in MEGA X and exported to IQ-Tree. The tree was generated using substitution model VT + F + G4 as a model of rate heterogeneity and Ultrafast Bootstrap with 1000 replicates. The bold IDs show those genes which have no repeat sequences. The bars on the tree nodes represent the frequencies of the amino acids. Orange = Clad 1; subclad (i) = monocot subclad containing H, D-rich, P, K, E-rich, and P, T, Y-rich domains, subclad (ii) = *SbSlp1* homologs in known Si accumulators, light blue = Clad 2, and grey = Clad 3.

A search employing *SbSlp1* as a query resulted in 342 protein sequences. We calculated the pI (7.03–10.34) and Mw (7135–74,345.9) along with the prediction of cleavage site and subcellular localization. Since positively charged amino acids are involved in biological silicification, we discarded all the proteins with a predicted pI value less than seven [21]. We also rejected the proteins that lacked signal peptide, as a silicifying protein must be secreted outside the silica cell membrane for biosilicification to take place in the paramural space. We also removed those peptide sequences for which we did not find the cleavage site. This resulted in the selection of 225 protein sequences. These sequences belonged to different gene families i.e., pistil-specific extension-like proteins, pollen Ole e 1 allergen and extensin family proteins, proline rich proteins (PRP), and some uncharacterized proteins. The ML tree was divided into three clads i.e., 1, 2, and 3. The first two clads had genes belonging to pollen Ole e 1 gene family while the third clad had mostly genes belonging to PRP family and some pollen ole e 1 members. Interestingly, all the genes which formed a clad with the *SbSlp1*, were pollen Ole e 1s. Monocot and dicot genes grouped in separate clads i.e., 1 and 2, respectively (Figure 3).

We then looked for the presence of repeats and found 17 genes without any repeats (bold in the tree) (Supplementary Table S3). The reported peptide sequence for in vitro silica precipitation is KEKPVKPPKKHPPP [21], therefore we search for the presence of this signature sequence in the identified homologs. A total of five genes in *SbSlp1* subclad i.e., *Brasy3G125000*, *Brahy.S03G0097200*, *Brast03G088000*, *Misin01G238500*, and *SeVir.9G291600* did not contain the H or D-rich regions or the consensus sequences KKPXPXKPKPXPKPXPXPX. The remaining genes in this subclad (i) had the H, D-rich, P, K, E-rich, and P, T, Y-rich domains, which were repeated more than once (Supplementary Table S3; Supplementary Figure S3).

Interestingly, we did not find the rice and other known high Si accumulators such as wheat that grouped closely with *SbSlp1*. Consequently, we checked wheat and rice homologs for the presence of consensus domains/sequences. Particularly, the rice *SbSlp1* homologs formed a separate subclad (ii) in Clad 1. The homologs of *B. staci*, *B. sylvaticum*, *B. hybridum*, were grouped together, while the wheat homologs were grouped together along with the one *H. vulgare* gene. Therefore, we aligned the genes in subclads (i and ii) to see the conserved/consensus sequences (Supplementary Figure S3a). The genes from clad-1 (ii) showed the presence of the P, K, E-rich region and H, D-rich domains. A total of five members of clad 1 (ii) belonging to *B. hybridum* and *B. staci*, and *B. distachyon* had three repeats of these regions. We noticed that the repeated sequences were followed by a G rich motif consisting of two or three Gs. One common feature of all the homologs containing these motifs was that before the consensus region, an H, D-rich domain was present and immediately after the consensus region a P, T, Y-rich domain was present similar to the work on SbSlp1 [21]. In clad 1 (i), this arrangement was repeated almost five times in the homologs with some modifications (Supplementary Figure S3b). While for clad 1 (ii) members, the arrangement of the above-mentioned domains was repeated only once with the exception of the five members of the clad 1(ii) mentioned above.

Now that we knew the conserved features of *SbSlp1* protein, we wanted to know if this feature was also present in the moderate-accumulators and low(non)-accumulators. For this, we selected known Si accumulators (rice, wheat, and sorghum), Si-intermediate types (soybean, cucumber) and low Si-accumulator (tomato) homologs. These homologs belonged to PRP proteins and did not contain the three domains that were found in Si-accumulators. Since, pollen Ole 1 allergens are present in other plants too (i.e., low-accumulators and moderately accumulators) [49], therefore, we searched for Arabidopsis pollen Ole 1s against *SbSlp1* but did not find the conserved domains. However, in a separate study against soybean genome (data not shown), an extension-like protein repeat containing soybean gene *Glyma.09G092700* was found. A search for repeats showed the presence of a domain (KPPIYKPPVYTPPVYKPPVEKPP) that was repeated eight times. By using *Glyma.09G092700* as a query gene to search against the known low (non) Si accumulators and moderate accumulators, the tomato genome did not result in any homologs even though its genome has PRP genes [50]. We used *TPRP1-F1* (a tomato PRP gene) to search for any homologs in the tomato genome. The results showed the members of seed storage/bifunctional inhibitor/lipid transfer proteins and hydrophobic seed proteins. However, none of the genes showed the domains of interest. As far as soybean and Arabidopsis PRPs are concerned, all the genes had repeats. However, two genes *Glyma.09G092700* and *At4g38770* had P, K, E-rich repeats (Supplementary Figure S3c). These observations suggest that moderate Si accumulators have the P, K, E-rich domain but they lack the other two domains present in Si-accumulators. Based on these observations, it could be proposed that at least these three sequences are required for biosilicification in Si-accumulators.

### 3.2. Silica Precipitation Potential of Repeats

**Figure 4:**
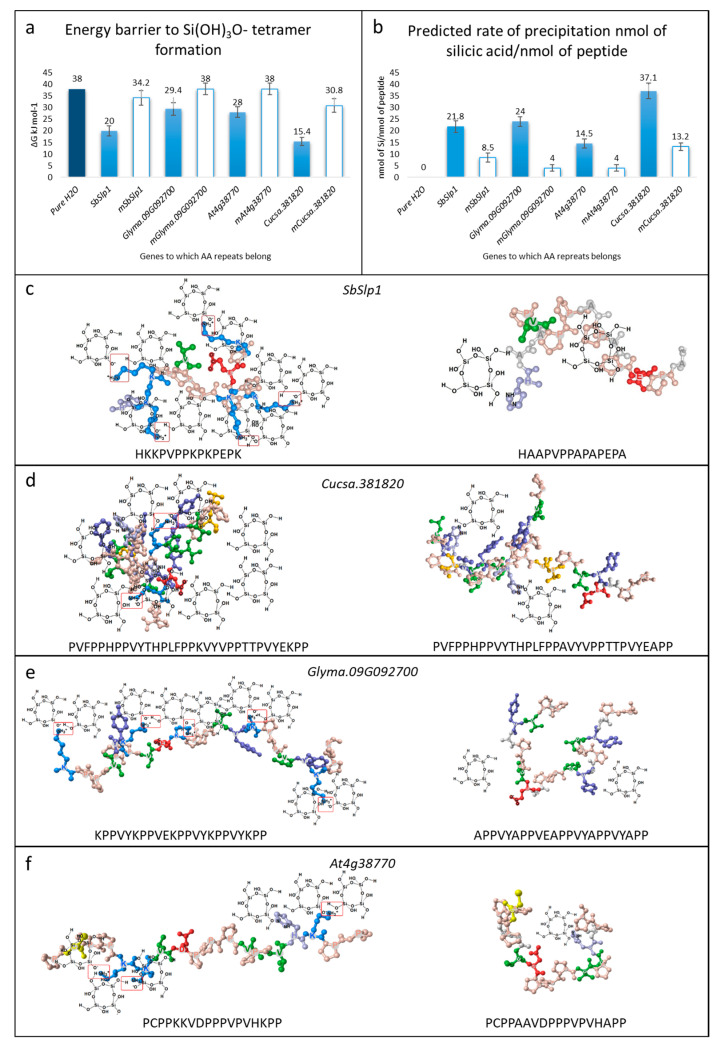
Silica precipitation potential of AA repeats in *SbSlp1*, *Cucsa.381820*, *Glyma.09G092700*, and *At4g38770*. (**a**) Energy barrier for Si(OH)_3_O^−^ tetramer formation (∆G kJ mol^−1^), (**b**) predicted rate of precipitation nmol of Si/nmol of peptide, and the interaction of (**c**) *SbSlp1*, (**d**) *Cucsa.381820*, *Glyma.09G092700*, and *At4g38770* peptides with silicic acid to form Si(OH)_3_O^−^ tetramer. The first panel of the figures **c**–**f** shows normal sequences and the second panels show the peptides where K was replaced with A. The letter “m” before the genes names in panels **a**,**b** represents the modified peptides.

We studied the molecular interactions and performed in-silico alanine scanning on the repeats present in *SbSlp1*, *Glyma.09G092700, Cucsa.381820*, and *At4g38770*. The computation tool Ligplot implemented in MOE allowed us to understand the silica polymerization potential of the selected AA repeats. When K is replaced with A, the energy barrier to Si(OH)_3_O^−^ tetramer formation increases (Figure 4a). With this increase in the energy barrier, the predicted rate of silica precipitation decreases (Figure 4b). The prediction suggested that the *SbSlp1* AA repeat, when mutated K to A, still was able to precipitate silica. This prediction is in accordance with the observation by Kumar et al. [19], that the same peptide was able to precipitate silica in a gel-like material (Figure 4c). Similarly, the changes in *Cucsa.381820* repeat showed decrease in the predicted rate of polymerization. *Glyma.09G092700* and *At4g38770* repeats showed limited efficiency to form Si(OH)_3_O^−^ tetramer (Figure 4d,e).

### 3.3. Digital Expression of SITs and Slps

**Figure 5:**
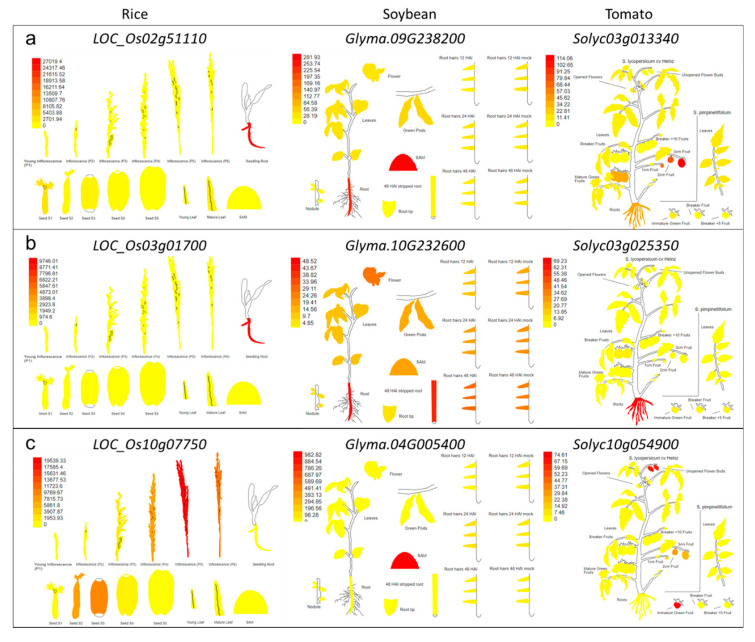
Digital gene expression of selected (**a**) channel type SIT, (**b**) Lsi2, and (**c**) Slp1 genes in rice, soybean, and tomato. The scale for each gene represents the Fragments Per Kilobase of transcript per Million mapped reads. Data analysis and graphical presentation was made by using ‘eFP browser’ of BAR tools (The Bio–Analytic Resource for Plant Biology, http://bar.utoronto.ca/).

As it is known that channel type SITs and Lsi2s are expressed mainly in roots and stem [4], while the Slp1 is expressed in inflorescence and leaves [19], we therefore studied their digital expression. We selected three known species for their potential to accumulate Si i.e., rice (Si-accumulators), soybean (moderate Si-accumulators) and tomato (low-accumulator) [51]. Rice and soybean channel type SITs strictly expressed in roots. However, in the case of tomato, the expression was lower in roots as compared to fruits (Figure 5a). For Lsi2s homologs, all three species showed high expressions in roots with soybean gene was also expressed in other plant tissues (Figure 5b). The putative Slp1s expression was maximum in rice inflorescence. While in case of soybean, the gene expressed in SAM (shoot apical meristem), and in tomato the genes were expressed in fruits (Figure 5c).

### 3.4. Co-Expression Networks of Si Transporters and Slps

**Figure 6:**
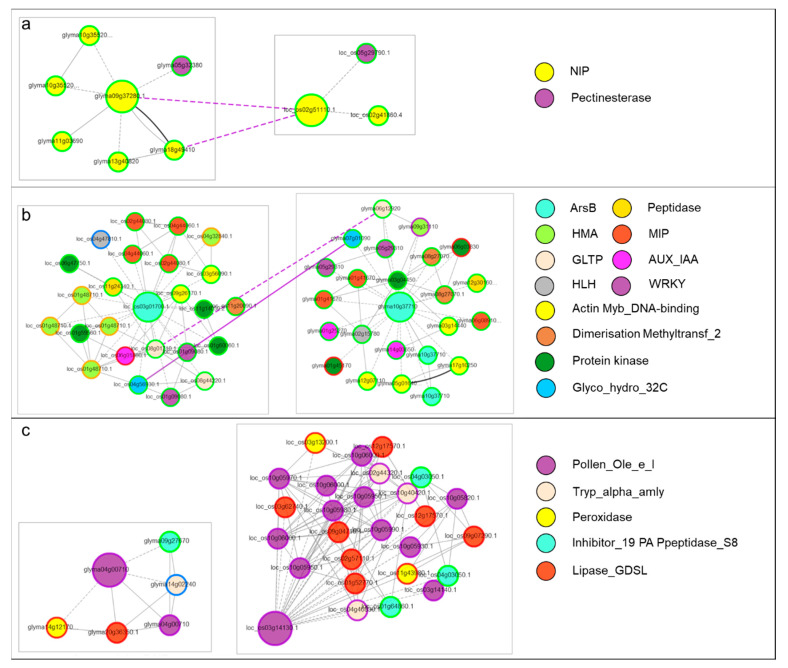
Monocot (rice) and dicot (soybean) gene coexpression networks of (**a**) channel type SITs, (**b**) Lsi2s, and (**c**) putative *Slp1s*. The solid edges show duplication while the dotted edges show speciation. The genes with the same shape and color belong to the same gene family and/or have pfam domains in common. Node borders indicate the phylostratum of the gene i.e., green (green plants), red (land plants), light blue (vascular plants), orange (monocot/dicots), brown (rosids/brassicales/malvids), and black (genus-specific phylostratum). The key on the right shows the gene family. The detail on the genes in the network is given in Supplementary Table S4.

To look for the coexpressed genes, we developed coexpression networks of selected genes in a dicot and a monocot species. We compared the gene networks of rice and soybean by using *Glyma0937280* and *LOC_Os02g51110*. The network comparison showed that speciation occurred in both lineages. Furthermore, the genes showed that other aquaporins and pectinesterase coexpressed with channel type SITs (Figure 6a). By comparing the gene modules of Lsi2s of rice and soybean, we found that genes related to cell wall modification as well as signaling were coexpressed. We also found that both duplication and speciation occurred in both lineages (Figure 6b). Finally, the coexpression networks of Slp1 homologs of rice and soybean showed that peroxidases, peptidases, and lipases coexpressed with these pollen Ole e 1s (Figure 6c).

## 4. Discussion

Channel type SITs that belong to NIP-III subfamily of aquaporins are thought to be evolutionarily restricted to monocots and sedges (known to be high Si accumulators), and early-diverging lineages (e.g., Sellaginellaceae and Equisetaceae) [18]. Furthermore, it is known that only a limited number of species from angiosperms have NIP-IIIs, and those which do have, show variation in the spacing between NPA motifs [1]. No detection of GSGR/STARG channel type SITs in Chlorophyta in our results suggests that the species explored in this research are either low Si-accumulators or their genomes contain other types of Si transporters. Previously, it is known that some Chlorophyte taxa i.e., *Tetraselmis pediastrum*, *Hydrodictyon* and some golden-algae accumulate Si in their cell walls [52,53,54]. Additionally, a recent study reported the presence of NIP family members in green algae however, the reported species did not contain STAR/GSGR SF, instead the authors found FAAR SF containing NIPs. Furthermore, the FAAR SF containing Chlorophyte NIP6:1 (*Klebsormidium nitens*) was unable to transport Germanium [17]. Therefore, the absence of channel type SITs from Chlorophyta may suggest that the NIPs evolved the Si transport-related features after the split of viridiplantae into Chlorophyta and Streptophyta or latter during the evolution of seed plants [55]. However, the observation that TIPs, PIPs, and MIPs were present in these species suggests this gene superfamily is omnipresent in these viridiplantae members. These observations are in agreement with the recent work by Pommering et al. [17] that the ability to transport Si could have arisen (due to subfunctionalization and neofunctionalization of arsenic efflux transporters into essential and beneficial plant nutrient transporters) later during the evolution of seed plants. Considering the focus of our study i.e., Lsi1 and Lsi2 homologs, the possibility of the presence of other types of transporters is not discussed here. Additionally, more specific studies on the Si concentrations could give clues about the classification of Chlorophyte taxa or species as Si accumulators, moderate Si-accumulators, and low Si-accumulators. All of channel type SITs in our study were found in Mangoliopsida (Supplementary Table S1). Current results are clearly in agreement with earlier reports that channel type SITs are widespread in monocots, since we found a higher number of channel type SITs in all studied members of Poaceae (Table 2). However, not all monocots included in our study have two NPA motifs with 108/109 AAs between the two motifs, for example, *Z. marina*, *A. comosus*, and *A. officinalis* channel type SITs homologs did not show these characteristic features known for the ability to transport Si (Supplementary Table S1). In dicots species belonging to *Brassicaceae* and *Solanaceae* families, we did not find channel type SITs having GSGR/STAR SF and defined NPA to NPA spacing. These observations are relevant to the fact that these species show very limited Si accumulation in planta [56]. However, a recent report in tomato suggested that a spacing of 109 AAs can still be functional when expressed in rice plant and *Xenopus* oocytes [9]. Other studies have also reported the absence of NIP-III members in this plant family [1,17]. Nonetheless, we did find NIP-III members (e.g., in tomato and papaya) but in both cases, the NPA to NPA spacing was 109 AAs (Figure 1). Another member of *Solanaceae* i.e., *Nicotiana tabaccum* has also been reported to have a functional Lsi1 homolog with two NPA motifs and a precise spacing of 108 AAs between the two NPA motifs [57]. Other dicot families i.e., *Myrtaceae*, *Rutaceae*, *Linaceae*, and *Oleaceae* also showed the absence of characteristic channel type SITs (Table 2), suggesting that the studied species are probably low Si-accumulators. Nonetheless, Si-transport inability is not universal in dicots and some species might have evolved reduced Si transport and absorption ability due to neofunctionalization as suggested in a recent study [17,58]. The interspecific variation of presence or absence and a variable number of channel type SITs and SF is interesting. Particularly, *H. annuus* which accumulates >1% (252–10,909 mg/Kg of dry weight; [59]) is possibly due to the presence of a higher number of channel type SITs (Table 2). The presence of two different types of SFs in two different families of Caryophyllales i.e., *C. quinoa* (*Chenopodiaceae*) and *A. hypochondriacus* (*Amaranthaceae*) suggest that modern plants (with an ability to accumulate Si) have evolved two different types of channel type SIT i.e., with STAR and GSGR SFs (Figure 1a). We state this because of the observation that STAR SF is present in species not restricted to few genera. For example, the earlier reports presented that only Equisetales (e.g., *E. arvense*) have STAR SF [46]. Our statement is consistent with a recent study by Deshmukh et al. [1], where authors also reported the presence of STAR SF in fern (*Mapania palustris*) and monocots (*Dipteris conjugate* and *Lepidosperma gibsonii* from Cyperaceae). Nevertheless, the presence of STAR SF in fern, monocot, and dicot clearly explain the presence and evolution of two SF in Lsi1s. This would be an interesting question for future studies targeting the evolution of aquaporin SFs in plants.

A very limited knowledge is available on the evolution of Lsi2s mainly because they do not show any sequence homology with the channel type SITs [4]. However, they are similar to arsenite Lsi2s that are present in bacteria and Archea [6]. Considering this i.e., a generalized metalloid transport capacity because of molecular mimicry from the evolutionary perspective, it is very much clear that these genes are widespread in Eukaryotes [18,60]. Our results showed that *OsLsi2* homologs were found in 71 viridiplantae species both from Streptophyta and Chlorophyta which confirms that Si transport is widespread in Viridiplantae. Moreover, together with previous reports (Table 2) [4,16], it could be stated that Si transport is an ancient feature. This is consistent with the findings of Marron et al. [16], where they reported that plant Lsi2s formed a separate clad with other Eukaryotic Lsi2s or Lsi2-like genes. As we said earlier, no sequence homology has been reported with channel type SITs, therefore, we did not find any conserved features of these genes to principally differentiate if a candidate gene could be dedicated to Lsi2s only or might have both Si and arsenite efflux capability like reported in rice. However, we could at least find a conserved motif in reported monocot Lsi2 homologs and the ones we report (Figure 2D) but its functional importance for monocots needs further investigation. Based on our findings, we could state that Lsi2s evolved distinctly i.e., two types of Lsi2s after the emergence of Polypodiopsida [16]. This assumption is based on the fact that all the Lsi2 homologs from Polypodiopsida, Lycopodiopsida, Sphanopsida, Bryopsida, and Marchantiopsida are grouped together (Supplementary Figure S2) [8].

The presence of Slps across Eukaryotic lineages is known mainly from the origin of the Si requirement in diatoms [20]. However, scarcity of the known Slps in plants has not enabled us to fully understand this process and the putative proteins in plants that take part in silica deposition [2]. Our knowledge of the plant lineages having the potential to accumulate Si has increased to a greater extent, thanks to the discovery of SITs by JF Ma and others [4,5,6,10,56]. However, we still lack the basic understanding of the gene/proteins involved in the biosilicification process in planta. In this work, we found the homologs of *SbSlp1* in almost all Streptophyta species (Table 2). Despite the inability of many plant species to accumulate large concentrations of silica in planta, where low Si-accumulator plants have shown extremely low biosilicification (and accumulation of phytoliths) in their cells, the homologs of *SbSlp1* are present in all Streptophyta species [2,4]. Nevertheless, the detailed investigations on the types of proteins for biosilicification in plants are scarce, and a complete picture of the evolution of these putative proteins will remain an open subject. However, considering the fact that *SbSlp1* is a member of pollen allergen Ole e 1 gene family, studies have confirmed that pollen allergens are present in green algae to angiosperms, where they expanded in angiosperms through multiple rounds of duplication and changes in polyploidy levels [49]. This is also true regarding gene structure evolution since, we found that in so-called Si accumulators, the H, D-rich, P, K, E-rich, and P, T, Y-rich domains are present in multiple repeats (Supplementary Figure S3). Meanwhile, so far, two studies have demonstrated the ability of a cationic PRP protein from cucumber and a Slp1 from sorghum to deposit silica in the cell wall and paramural space, respectively [19,25]. Therefore, based on the observations on putative Slps in our study and structures reported earlier, it cannot be necessarily said that the process of biosilicification needs the presence of H, D-rich domain and/or P, T, Y-rich domain. Regardless of the fact that biosilicification has been reported in soybean (i.e., the presence of phytoliths) [61], we did not find H, D-rich domain and P, T, Y-rich domain in the soybean *SbSlp1* homologs found in our BlastP search (Supplementary Table S3; Supplementary Figure S3c). This further strengthens the less important role of both domains in biosilicification. The lack of P, K, E-rich domain from non-accumulators, and its presence in moderate-accumulators and Si-accumulators suggests the essential role of this domain in biosilicification process. However, based on the study by Kauss et al. (2003), due to the presence of a high content of positively charged AAs in three peptides tested by their team, it can be concluded that positioning of K along with R as clusters or partially adjacent positioning did not clearly disturb the ability of peptide to precipitate silica. Our results showing the changes in the energy barrier and the predicted rate of precipitation of silicic acid/nmol of peptide also suggest that replacing K with A increases the energy barrier and reduced the rate of precipitation (Figure 4). The authors concluded that silica deposition is mainly dependent on a sufficiently high density of positively charged amino acids than on a peptide’s primary sequence.

The Si deposition benefits plants by increasing plants’ ability to withstand biotic and abiotic stresses; hence considered as biostimulant in agriculture [62]. However, to benefit from Si, plants must uptake transport, and accumulate/deposit it in different plant tissues at physiological conditions with temperatures ranging from 0 to 40 ℃, neutral pH, and ambient pressure [14]. This marvelous process is completed by the above discussed SITs i.e., channel type SITs and Lsi2s and many uncharacterized Slps as well as many unknown players in this process [21]. To complete the process of biosilicification, plants may mobilize Si from roots through the expression of channel type SITs and Lsi2s in roots and stems [4]. The expression of these SITs varies in different species [2]. Our digital expression results in rice, soybean, and tomato greatly complement that channel type SITs are expressed in roots (Figure 5a). However, the homologs of Lsi1 in rice, and maize i.e., Lsi6s have been reported to be expressed in stem as well [63]. The Lsi2s are mainly expressed in rice in roots, where these genes make a cooperative network to move Si within roots [6]. Interestingly, we also found that the putative Lsi2s in three species differing in Si accumulation capacity express in roots (Figure 5b). The understanding of the mechanism of Si transport has been now well developed, however, emerging data of the interactome helping researchers understand how Si interacts with other genes and proteins to help plants against biotic and abiotic factors. The coexpression of channel-type SITs with pectinesterases is interesting because the level and pattern of pectin esterification play a role in constitutive resistance to many fungal and bacterial pathogens influencing the susceptibility of plant cell wall to microbial pectin-degrading enzymes [64,65]. Apart from this, it was recently suggested that a *B. distachyon* mutant exhibits a range of alterations in the composition of non-cellulosic polysaccharides i.e., monosaccharides associated with pectins (rhamnose, galacturonic acid, and galactose) [66]. This study and our in-silico coexpression results suggest that transport of Si via Lsi1, when disrupted, could affect the cell wall composition. There is an increasing body of literature on the role of cell wall modification related genes’ involvement in multiple abiotic and biotic stress related processes [67,68,69]. Furthermore, the coexpression of Lsi2s with known transcription factors, i.e., WRKYs, is quite understandable since WRKYs are known for their ability to help plants against abiotic stresses [70]. However, so far, the combined role of Si and WRKYs has not been explored in plants and would be an interesting topic for future research. In addition to WRKYs, the coexpression of peptidase, protein kinases and AUX-IAA is possibly due to the fact that silicic acid is involved in signal transduction pathways in plant defenses against microbes [71,72,73]. Previously, Vatansever et al. [11] also reported such coexpression between SITCs and resistance signaling related genes. Finally, the expression of putative rice and tomato Slp1 in seed and inflorescence, and fruit suggests that these proteins have additional roles (Figure 5c) i.e., important physiological roles in pollen, especially the pollination process [49,74]. This is consistent with the findings of Kumar et al. [19] i.e., the expression of *SbSlp1* was detected in immature leaves and inflorescence. Additionally, the PRP in cucumber was expressed in cell walls [25], and it is known that biosilicification in plants is associated with cell wall polymers [75], particularly in plants having resistance to diseases [69]. Our results also confirmed the coexpression of arabinogalactan proteins (Figure 6c). Hence, in view of these reports, Si seems to increase plants’ ability to withstand against different stresses by coexpressing with structural integrity and signaling related genes. Together, these results enhance our understanding about the mechanism of biosilicification in plants and lead us towards many interesting questions to be answered.

## 5. Conclusions

Together, our results demonstrate that early Viridiplantae lineages, i.e., green algae, lack characteristic channel type SITs and likely have other mechanisms facilitating Si influx. Furthermore, these genes evolved two different types of SFs i.e., STAR and GSGR. Additionally, these genes are not strictly restricted in monocot species and can be found in a wide range of dicot species. Two types of Lsi2s evolved after the emergence of Polypodiopsida. However, considering the presence of Lsi2 outside Streptophyta i.e., Chlorophyta, it could be stated that Lsi2s are ancient. Similar to channel type SITs, the Slp1 homologs were not found in Chlorophyta. High number of repeats of P, K, E-rich domain in monocots (or Si accumulators) in addition to two other conserved domains could be the key feature behind the presence of relatively larger Si quantities in these plants. The presence of P, K, E-rich domain within Si-accumulators and moderate-accumulators suggests its functional importance for the biosilicification process.

## Figures and Tables

**Table 1:** The 80 Viridiplantae species and respective genome versions used in this study.

Species	Genome Version	Species	Genome Version
*Amaranthus hypochondriacus*	V2.1	*Kalanchoe laxiflora*	V1.1
*Amborella trichopoda*	V1.0	*Lactuca sativa*	V8
*Anacardium occidentale*	V0.9	*Linum usitatissimum*	V1.0
*Ananas comosus*	V3	*Malus domestica*	V1.0
*Aquilegia coerulea*	V3.1	*Manihot esculenta*	V6.1
*Arabidopsis halleri*	V1.1	*Marchantia polymorpha*	V3.1
*Arabidopsis lyrata*	V2.1	*Medicago truncatula*	4.0V1
*Arabidopsis thaliana*	TAIR10	*Micromonas pusilla* CCMP1545	V3.0
*Asparagus officinalis*	V1.1	*Mimulus guttatus*	V2.0
*Boechera stricta*	V1.2	*Miscanthus sinensis*	V7.1
*Botryococcus braunii*	V2.1	*Musa acuminata*	V1
*Brachypodium distachyon*	V3.1	*Olea europaea Var. sylVestris*	V1.0
*Brachypodium hybridum*	V1.1	*Oropetium thomaeum*	V1.0
*Brachypodium stacei*	V1.1	*Oryza sativa*	V7_JGI
*Brachypodium sylVaticum*	V1.1	*Ostreococcus lucimarinus*	V2.0
*Brassica oleracea capitata*	V1.0	*Panicum hallii*	V3.1
*Brassica rapa FPsc*	V1.3	*Panicum Virgatum*	V4.1
*Capsella grandiflora*	V1.1	*Phaseolus Vulgaris*	V2.1
*Capsella rubella*	V1.0	*Physcomitrella patens*	V3.3
*Carica papaya*	V0.4	*Populus deltoides* WV94	V2.1
*Chenopodium quinoa*	V1.0	*Populus trichocarpa*	V3.1
*Chlamydomonas reinhardtii*	V5.5	*Prunus persica*	V2.1
*Chromochloris zofingiensis*	V5.2.3.2	*Ricinus communis*	V0.1
*Cicer arietinum*	V1.0	*Salix purpurea*	V1.0
*Citrus clementina*	V1.0	*Selaginella moellendorffii*	V1.0
*Citrus sinensis*	V1.1	*Setaria italica*	V2.2
*Coccomyxa subellipsoidea* C-169	V2.0	*Setaria Viridis*	V2.1
*Coffea arabica*	UCDV0.5	*Solanum lycopersicum* iTAG	2.4
*Cucumis sativus*	V1.0	*Solanum tuberosum*	V4.03
*Daucus carota*	V2.0	*Sorghum bicolor*	V3.1.1
*Dunaliella salina*	V1.0	*Sphagnum fallax*	V0.5
*Eucalyptus grandis*	V2.0	*Spirodela polyrhiza*	V2
*Eutrema salsugineum*	V1.0	*Theobroma cacao*	V1.1
*Fragaria Vesca*	V1.1	*Trifolium pratense*	V2
*Glycine max* Wm82	a2.V1	*Triticum aestiVum*	V2.2
*Gossypium hirsutum*	V1.1	*Vigna unguiculata*	V1.1
*Gossypium raimondii*	V2.1	*Vitis Vinifera*	12X
*Helianthus annuus*	r1.2	*VolVox carteri*	V2.1
*Hordeum Vulgare*	r1	*Zea mays*	Ensembl-18
*Kalanchoe fedtschenkoi*	V1.1	*Zostera marina*	V2.2

Available at Phytozome v12.1; Joint Genome Institute (JGI; https://www.phytozome.net) [27].

**Table 2:** Summary of Lsi1, Lsi2, and Slp1 homologs found in 80 studied Viridiplantae species.

Class	Order	Family	Species	Lsi1	Lsi2	Slp1
Trebouxiophyceae	Trebouxiophyceae	*Coccomyxaceae*	*Coccomyxa subellipsoidea*			
	Trebouxiales	*Botryococcaceae*	*Botryococcus braunii*			
Mamiellophyceae	Mamiellales	*Mamiellaceae*	*Micromonas PUSILLA ccmp1545*		1	
		*Bathycoccaceae*	*Osteococcus lucimarinus*		1	
Chlorophyceae	Sphaeropleales	*Chromochloridaceae*	*Chromochloris zofingiensis*		2	
	Chlamydomonadales	*Volvocaceae*	*Volvox carteri*		2	
		*Chlamydomonadaceae*	*Chlamydomonas reinhardtii*		1	
		*Dunaliellaceae*	*Dunaliella salina*		1	
Lycopodiopsida	Selaginellales	*Selaginellaceae*	*Selaginella moellendorffii*		3	
Sphagnopsida	Sphagnales	*Sphagnaceae*	*Sphagnum fallax*		1	1
Bryopsida	Funariales	*Funariaceae*	*Physcomitrella patens*		2	
Marchantiopsida	Marchantiales	*Marchantiaceae*	*Marchantia polymorpha*		1	
Liliopsida	Alismatales	*Araceae*	*Spirodela polyrhiza*		1	1
		*Zosteraceae*	*Zostera marina*			1
	Asparagales	*Asparagaceae*	*Asparagus officinalis*		1	2
	Zingiberales	*Musaceae*	*Musa acuminata*	4	4	1
	Poales	*Bromeliaceae*	*Ananas comosus*		4	4
		*Poaceae*	*Oropetium thomaeum*	1	4	3
			*Panicum halli*	1	3	4
			*Panicum virgatum*	3	8	8
			*Brachypodium stacei*	2	5	6
			*Brachypodium hybridum*	4	10	11
			*Brachypodium sylvaticum*	2	4	8
			*Brachypodium distachyon*	2	5	5
			*Zea mays*	4	5	4
			*Triticum aestivum*	3	6	20
			*Sorghum bicolor*	2	5	3
			*Setaria viridis*	2	5	5
			*Setaria italica*	2	5	4
			*Oryza sativa*	2	4	7
			*Hordeum vulgare*	3	5	2
			*Miscanthus sinensis*	4		10
Magnoliopsida	Ranunculales	*Ranunculaceae*	*Aquilegia coerulea*	1	1	2
	Myrtales	*Myrtaceae*	*Eucalyptus grandis*		2	2
	Saxifragales	*Crassulaceae*	*Kalanchoe laxiflora*	1	2	
			*Kalanchoe fedtschenkoi*		1	1
	Vitales	*Vitaceae*	*Vitis vinifera*	1	1	2
	Caryophyllales	*Chenopodiaceae*	*Chenopodium quinoa*	2	2	2
		*Amaranthaceae*	*Amaranthus hypochondriacus*	1	2	2
	Gentianales	*Rubiaceae*	*Coffea arabica*		1	3
	Amborellales	*Amborellaceae*	*Amborella trichopoda*	1	4	1
	Asterales	*Asteraceae*	*Lactuca sativa*	2	1	1
			*Helianthus annuus*	7	6	3
	Lamiales	*Lamiaceae*	*Capsella grandiflora*		1	5
		*Phrymaceae*	*Mimulus guttatus*	1	1	
		*Oleaceae*	*Olea europaea*		1	5
	Solanales	*Solanaceae*	*Solanum tuberosum*		1	2
			*Solanum lycopersicum*	1	1	1
	Apiales	*Apiaceae*	*Daucus carota*	1	1	3
	Fabales	*Fabaceae*	*Trifolium pratense*	1	3	1
			*Vigna unguiculata*		3	2
			*Phaseolus vulgaris*		2	2
			*Medicago truncatula*		2	1
			*Glycine max*	2	1	1
			*Cicer arietinum*	1		1
	Rosales	*Rosaceae*	*Fragaria vesca*	3	2	2
			*Prunus persica*	1	1	1
			*Malus domestica*	1	2	1
	Malpighiales	*Linaceae*	*Linum usitatissimum*		2	2
		*Euphorbiaceae*	*Ricinum communis*	1	1	
			*Mahihot esculenta*	1	1	1
		*Salicaceae*	*Salix purpurea*	1	2	3
			*Populus deltoides*	1	1	2
			*Populus trichocarpa*	1	2	2
	Cucurbitales	*Cucurbitaceae*	*Cucumis sativus*	1	1	2
	Brassicales	*Brassicaceae*	*Capsella rubella*		1	6
			*Eutrema salugineum*		1	6
			*Boechera stricta*		1	4
			*Brassica oleracea*		1	5
			*Brassica rapa*		1	6
			*Arabidopsis halleri*		1	2
			*Arabidopsis lyrata*		1	7
			*Arabidopsis thaliana*		1	6
		*Caricaceae*	*Carica papaya*	1	1	1
	Malvales	*Malvaceae*	*Theobroma cacao*	1	4	1
			*Gossypium raimondii*		3	2
			*Gossypium hirsutum*	2	3	4
	Sapindales	*Anacardiacea*	*Anacardium occidentale*	1	1	
		*Rutaceae*	*Citrus clementia*		1	3
			*Citrus sinensis*		1	2

## Supplementary Materials

The following are available online at https://www.mdpi.com/2223-7747/9/11/1612/s1, Table S1. List of homologs of rice channel type SITs in the studied plant genomes. Table S2. List of homologs of rice *Lsi2* gene in the studied plant species. Table S3. Siliplant1 homologs in the studied plant species. Table S4. Details on the genes in the coexpression networks of Lsi2s and Siliplant1s. Figure S1. (a) Sequence alignment of known efflux Si transporters from rice, maize, barley, cucumber, pumpkin, and an Arabidopsis gene. (b) The highly conserved motif in known Si efflux transporters. Figure S2. Maximum-likelihood tree of Si efflux transporters. ML tree of *OsLsi2* homologs in studied viridiplantae genomes (plus two Equisetum Lsi2s). *KU821730* (*Spongosphaera streptacantha* SIT-L gene) was used as an outgroup. The sequences were then aligned by MUSCLE in MEGA X and exported to IQ-Tree. The tree was generated using substitution model VI+I+G4 (Invar+Gamma with 4 categories) as a model of rate heterogeneity and Ultrafast Bootstrap with 1000 replicates. Figure S3. (a) Alignment of Slp1 homologs (sub-clad 1Cii) showing the conserved regions. (b) Alignment of *SbSlp1* homologs that clustered together (sub-clad 1Ci). (c) Repeats found in soybean and Arabidopsis PRP genes. Red bars = H, D-rich domain, Orange bars = P, K, E-rich domain, Green bars = P, T, Y-rich domain, and Pink = G-rich region. The sequences above the alignment in (a) represent the additional sequences present within genes 8-13 in alignment. The colors of the borders match with those inserted in the alignment. The position of the inserted boxes represents the position of the respective sequences.
