# Supplementary material for: In-silico Exploration of Channel Type and Efflux Silicon Transporters and Silicification Proteins in 80 Sequenced Viridiplantae Genomes
Source: Plants (Basel). 2020 Nov 20;9(11):1612. doi: 10.3390/plants9111612 (PMC7709012; doi:10.3390/plants9111612)
Supplement: Supplementary file 1 [file plants-09-01612-s001.zip › Supplementary Figures.docx]

**Supplementary Figures**


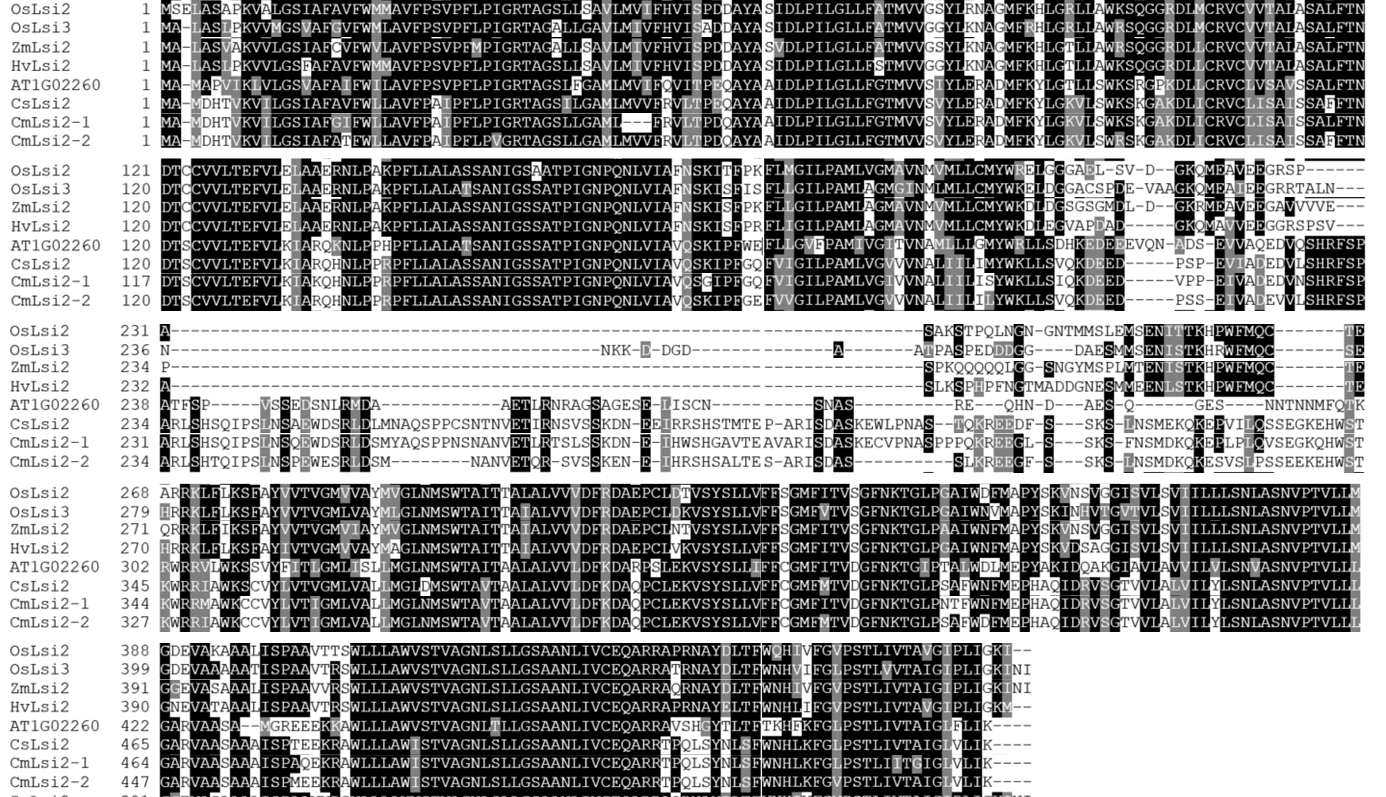


(a)


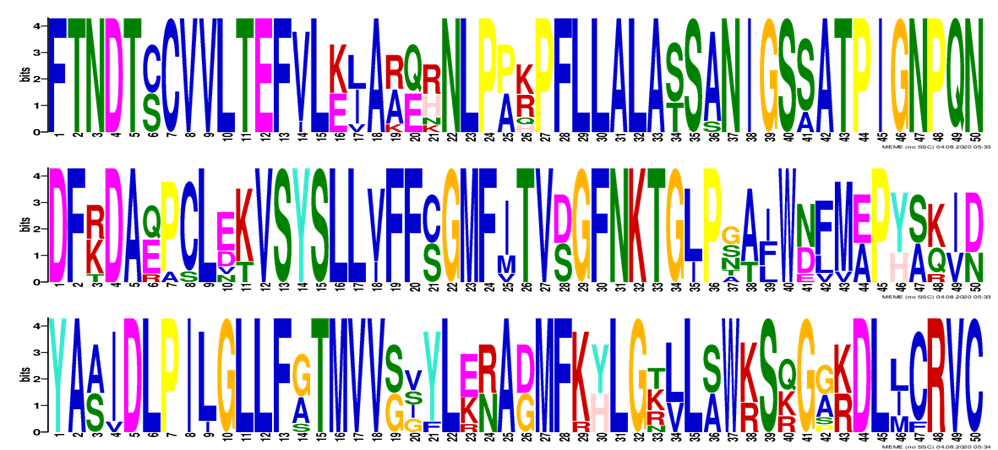


(b)

**Supplementary Figure S1.** (a) Sequence alignment of known efflux Si transporter from rice, maize, barley, cucumber, pumpkin, and an Arabidopsis gene. (b) The highly conserved motifs in known Si efflux transporters.


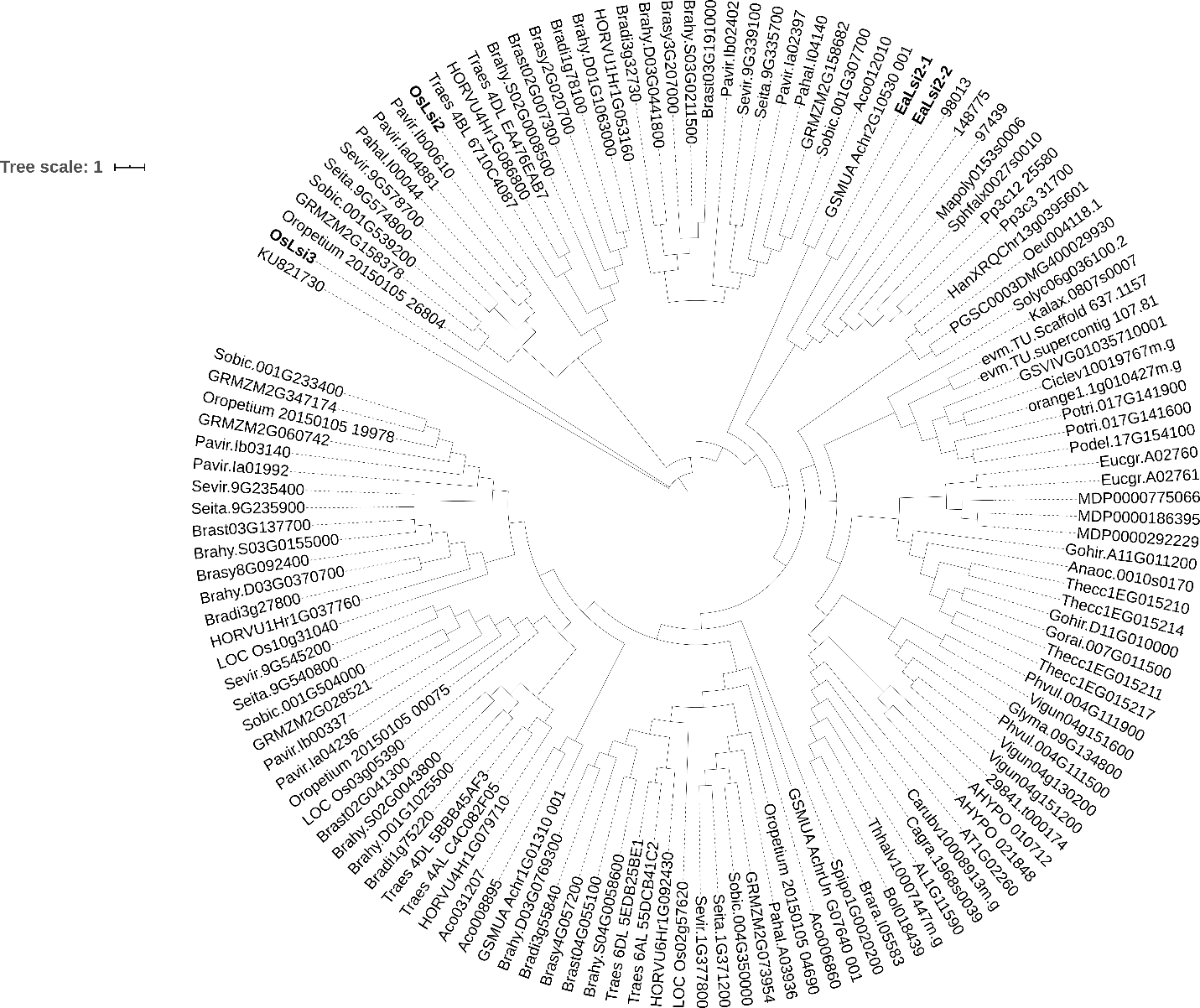


**Supplementary Figure S2.** Maximum-likelihood tree of Si efflux transporters. ML tree of *OsLsi2* homologs in studied viridiplantae genomes (plus two Equisetum Lsi2s). *KU821730* (*Spongosphaera streptacantha* SIT-L gene) was used as an outgroup. The sequences were then aligned by MUSCLE in MEGA X and exported to IQ-Tree. The tree was generated using substitution model VI+I+G4 (Invar+Gamma with 4 categories) as a model of rate heterogeneity and Ultrafast Bootstrap with 1000 replicates.


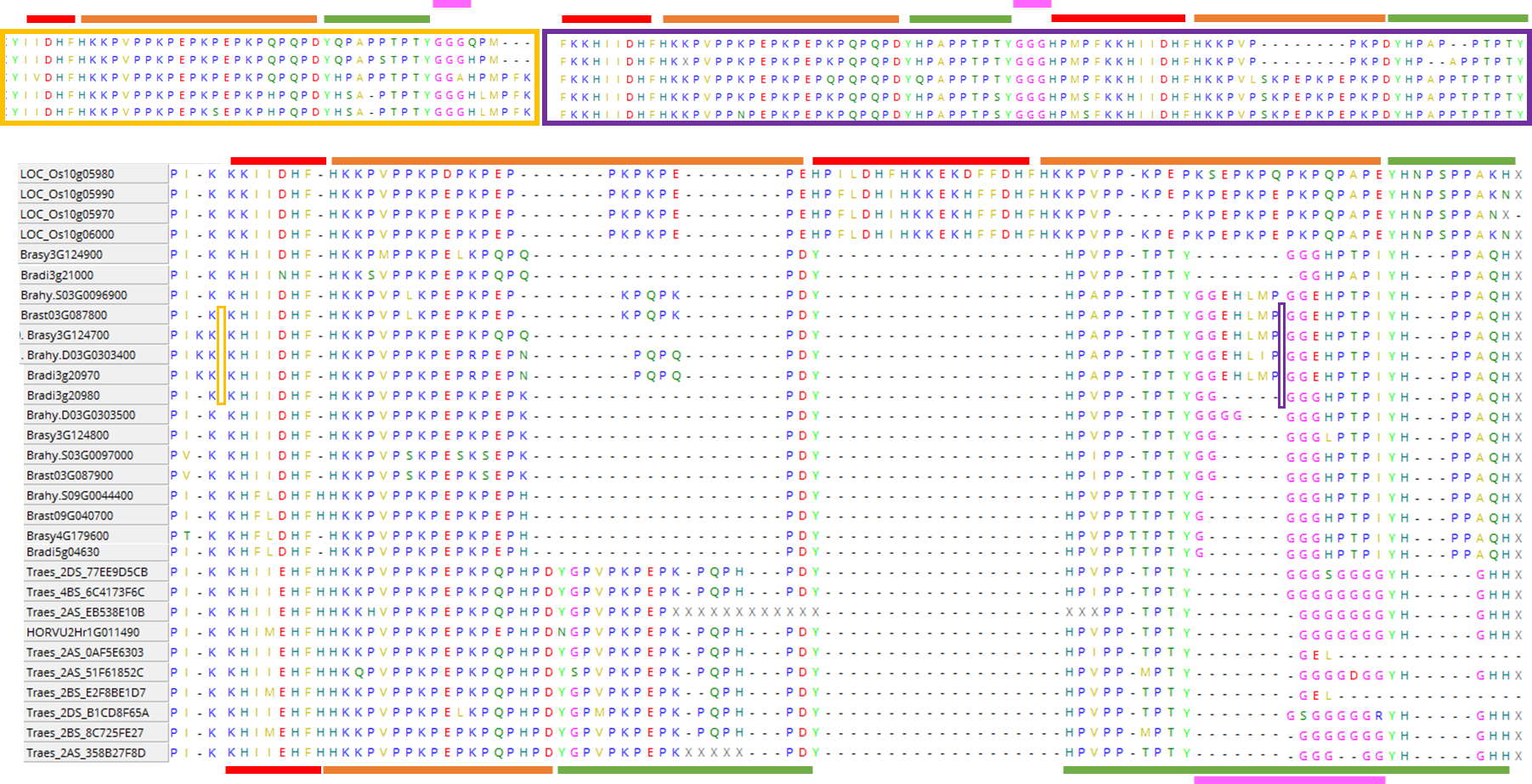


(a)


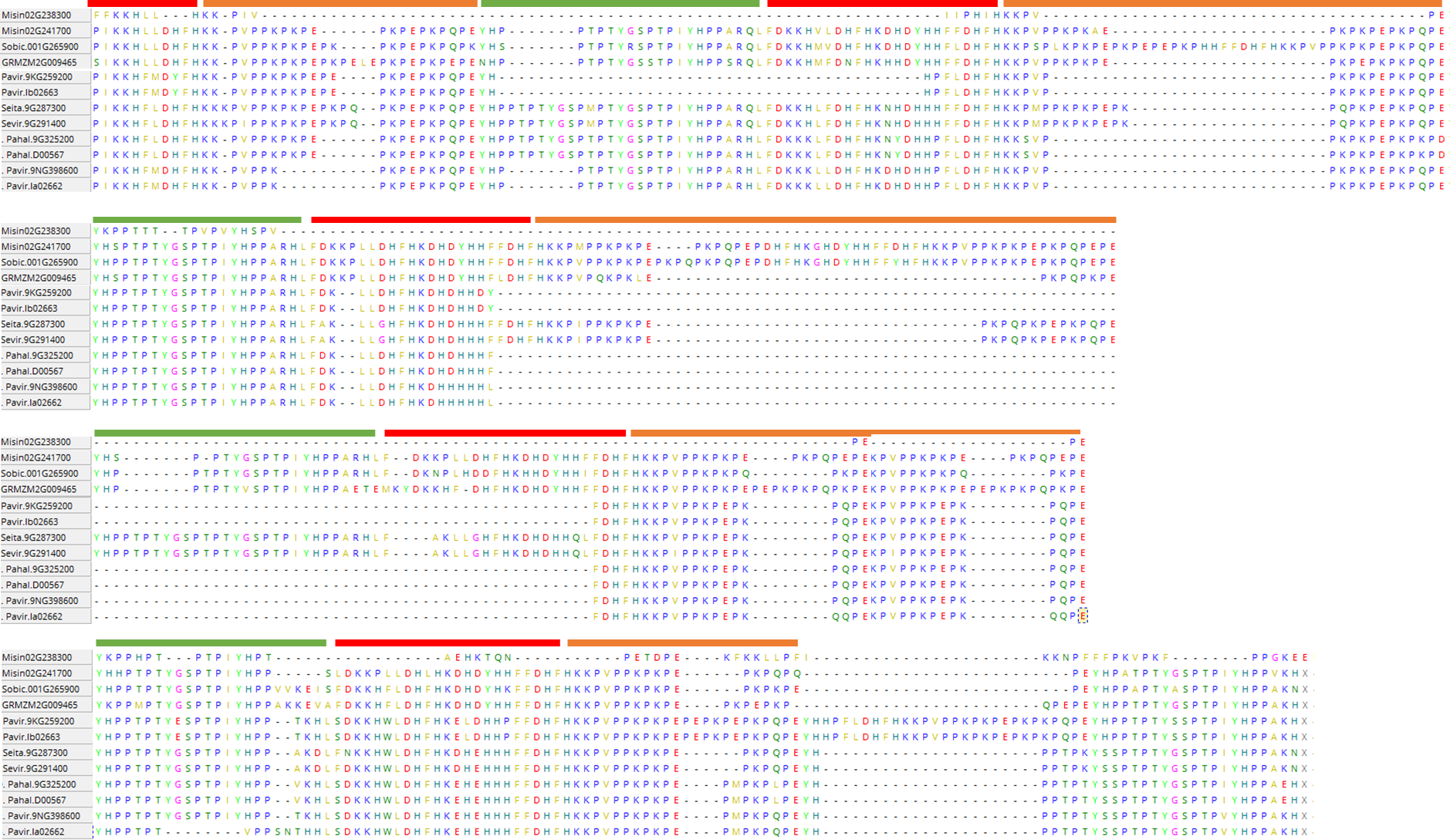


(b)


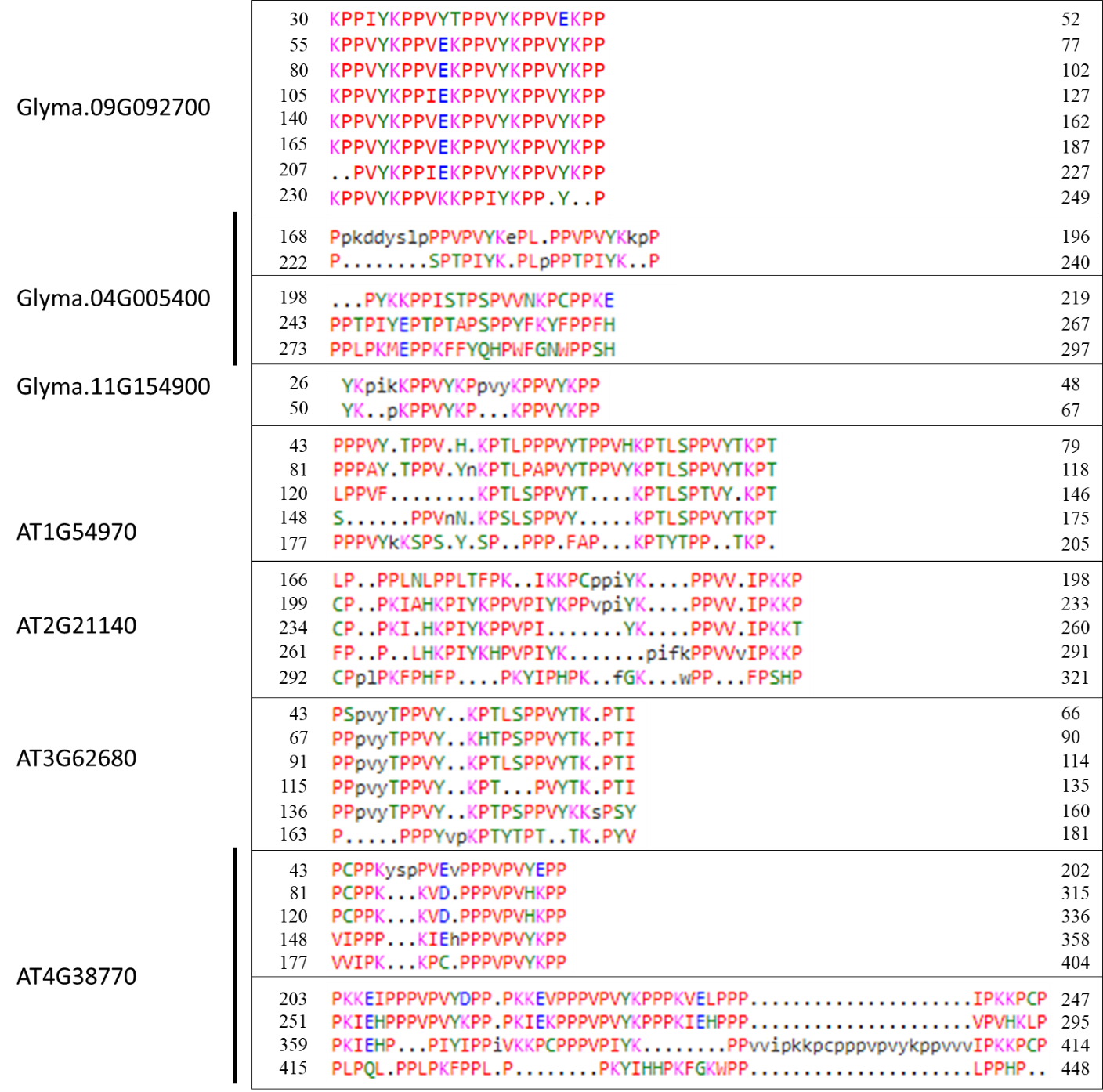


(c)

**Supplementary Figure 3.** (a) Alignment of Slp1 homologs (sub-clad 1Cii) showing the conserved regions. (b) Alignment of *SbSlp1* homologs that clustered together (sub-clad 1Ci). (c) Repeats found in soybean and Arabidopsis PRP genes. Red bars = H, D-rich domain, Orange bars = P, K, E-rich domain, Green bars = P, T, Y-rich domain, and Pink = G-rich region. The sequences above the alignment in (a) represent the additional sequences present within genes 8-13 in alignment. The colors of the borders match with those inserted in the alignment. The position of the inserted boxes represents the position of the respective sequences.
